# Structured report data can be used to develop deep learning algorithms: a proof of concept in ankle radiographs

**DOI:** 10.1186/s13244-019-0777-8

**Published:** 2019-09-23

**Authors:** Daniel Pinto dos Santos, Sebastian Brodehl, Bettina Baeßler, Gordon Arnhold, Thomas Dratsch, Seung-Hun Chon, Peter Mildenberger, Florian Jungmann

**Affiliations:** 1 Department of Radiology, University Hospital of Cologne, Kerpener Str. 62, 50937 Cologne, Germany; 2 Department of Informatics, University Mainz, Mainz, Germany; 3 Department of Radiology, University Medical Center Mainz, Mainz, Germany; 4 Department of Surgery, University Hospital of Cologne, Cologne, Germany

**Keywords:** Structured reporting, Workflow, Machine learning, Radiography, Ankle fractures

## Abstract

**Background:**

Data used for training of deep learning networks usually needs large amounts of accurate labels. These labels are usually extracted from reports using natural language processing or by time-consuming manual review. The aim of this study was therefore to develop and evaluate a workflow for using data from structured reports as labels to be used in a deep learning application.

**Materials and methods:**

We included all plain anteriorposterior radiographs of the ankle for which structured reports were available. A workflow was designed and implemented where a script was used to automatically retrieve, convert, and anonymize the respective radiographs of cases where fractures were either present or absent from the institution’s picture archiving and communication system (PACS). These images were then used to retrain a pretrained deep convolutional neural network. Finally, performance was evaluated on a set of previously unseen radiographs.

**Results:**

Once implemented and configured, completion of the whole workflow took under 1 h. A total of 157 structured reports were retrieved from the reporting platform. For all structured reports, corresponding radiographs were successfully retrieved from the PACS and fed into the training process. On an unseen validation subset, the model showed a satisfactory performance with an area under the curve of 0.850 (95% CI 0.634–1.000) for detection of fractures.

**Conclusion:**

We demonstrate that data obtained from structured reports written in clinical routine can be used to successfully train deep learning algorithms. This highlights the potential role of structured reporting for the future of radiology, especially in the context of deep learning.

**Electronic supplementary material:**

The online version of this article (10.1186/s13244-019-0777-8) contains supplementary material, which is available to authorized users.

## Key points

- Data from structured reports can greatly facilitate development of deep learning algorithms.
- Fully automated workflows for training of deep learning networks can easily be implemented.
- A proof of concept for the detection of ankle fractures is presented and achieves satisfactory performance.

## Background

Recently, the application of computer vision techniques and especially deep learning to evaluate plain radiographs or computed tomography exams has been extensively discussed in radiology [1–3]. Consequently, in the last few years, numerous groups have published papers describing promising applications of deep learning algorithms in radiology.

Various studies were reported where the authors developed and trained deep neural networks to perform automated diagnosis or triage of plain radiographs. While some of those relied on manual review and labeling of the images to establish a valid ground truth (e.g., detection for of humerus fractures [4], hip fractures [5], and wrist fractures [6]), other relied on automatically extracting image labels from the written radiological reports associated with the imaging study [7–9]. As radiological reports are usually written in a prose-like, non-standardized form, techniques such as natural language processing (NLP) are needed, to analyze the reports and extract meaningful labels to be used in further training of the neural network. Compared to manual review labeling, the latter approach is much more efficient and scalable, thus enabling larger datasets to be compiled for the subsequent training of the neural networks. However, as was shown, e.g., in the case of the CheXNet paper [10], this also has the potential to introduce inaccuracies and uncertainties which are inherent to variations in NLP [11].

With more and more advances in computer vision and deep learning technologies and algorithms, it seems that one of the only remaining challenges is the availability of accurately labeled datasets. It would, therefore, be desirable if data from clinical routine could be used to provide reliable labels without the need for potentially error-prone NLP or time-consuming manual labeling by human expert readers.

One possibility to make data from clinical routine more readily usable could be structured reporting (SR) which has long been proposed by various radiological societies [12–14]. Structured reporting aims at standardizing report content and language, thus making the report more machine readable. Some studies have demonstrated the usage of data extracted from structured reports for calculation of various statistics [15, 16].

This approach could also be useful in the context of training deep learning algorithms. Therefore, the aim of this study was to propose an example workflow where date from structured reports is used to extract accurately labeled training data from an institution’s picture archiving and communication system (PACS). As a proof of concept, we show this by using this data to retrain a pretrained convolutional neural network (Inception V3) for the detection of fractures in ankle radiographs.

## Materials and methods

Starting in late 2017, structured reporting was introduced at our tertiary care institution. Various IHE MRRT-compliant report templates were created and installed in a dedicated open-source reporting platform [17, 18]. The reporting platform had previously been developed at our institution using only standard web-technologies and could be accessed from the clinical workstations by the reporting radiologists. To facilitate its usage in clinical routine, it was fully integrated in the radiologists’ workflow and connected to the institutions radiology information system (RIS) and PACS. All radiologists received in-person training on how to use the reporting platform and the templates and could contact the developer any time if problems occurred. At the time of reporting, the radiologists were able to either use the standard RIS reporting engine, including speech recognition, or start reporting in the structured reporting platform. Usage of the reporting platform was neither enforced nor incentivized. To ensure the correct patient and study context, the RIS constructs a URL-call that passes the relevant patient and study information to the reporting platform. Upon completion of the radiological report in the platform data, the structured reports were stored in the platform’s database as discrete information thus allowing for easily machine-readable reports.

### Use case and patient selection

**Fig. 1:**
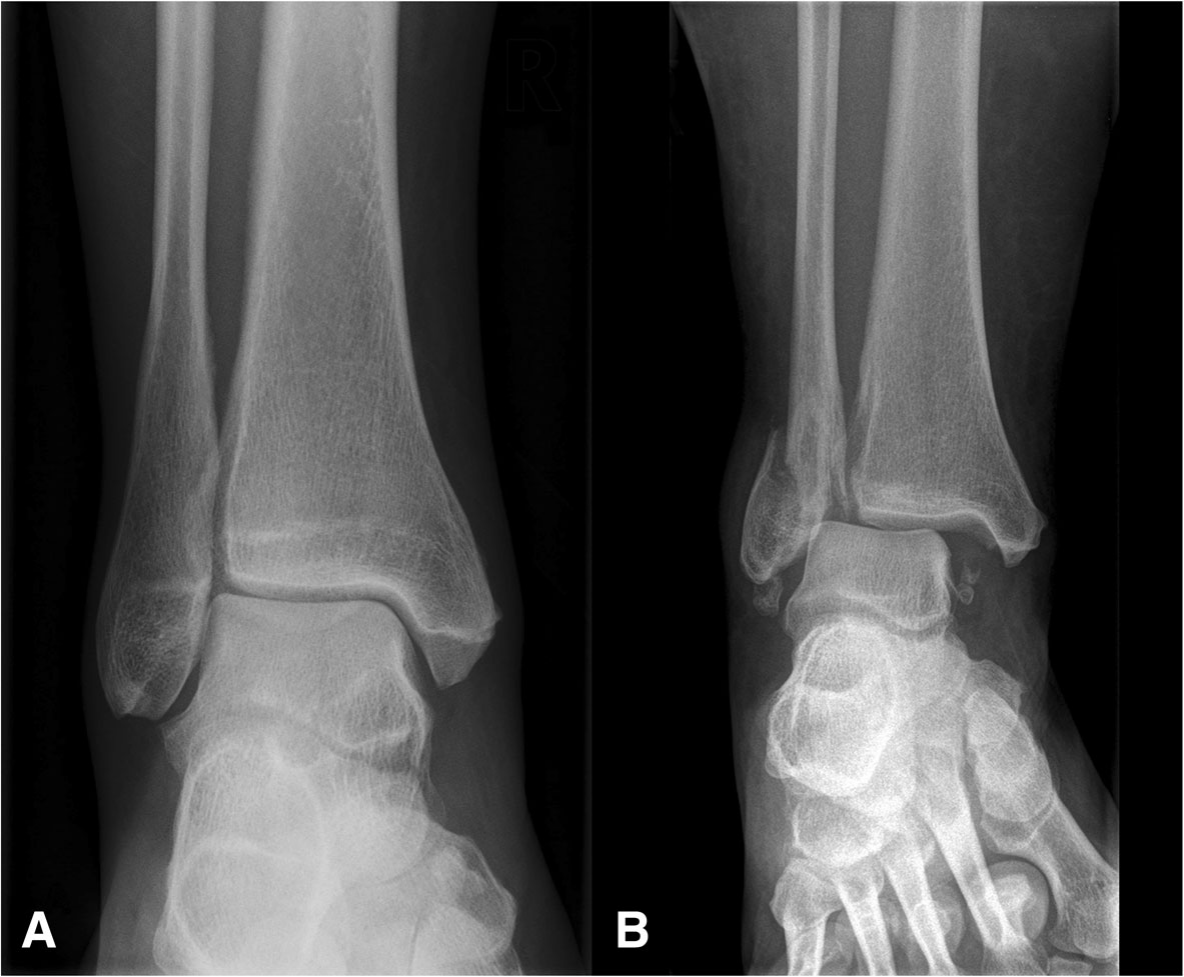
Examples of radiographs used in the study (**a** no fracture, **b** fracture present)

During the initial phase of set up of the structured reporting platform, various report templates had been created. While most templates focused on computed tomography or magnetic resonance imaging, some templates pertaining to conventional radiography were also developed. As basis for this proof of concept, we chose to focus on a rather simple use case using only plain radiographs. For the purpose of this study, we chose to use data from cases where plain radiographs of the ankle were obtained in the context of trauma (fracture/no fracture) and for which structured reports had been written using the above-mentioned platform (Fig. 1).

All reports were written between August 2017 and September 2018. As radiologists were free to decide whether to use the structured reporting template or to write a conventional narrative report, the studies included were not consecutive.

### Structured reporting and image retrieval

**Fig. 2:**
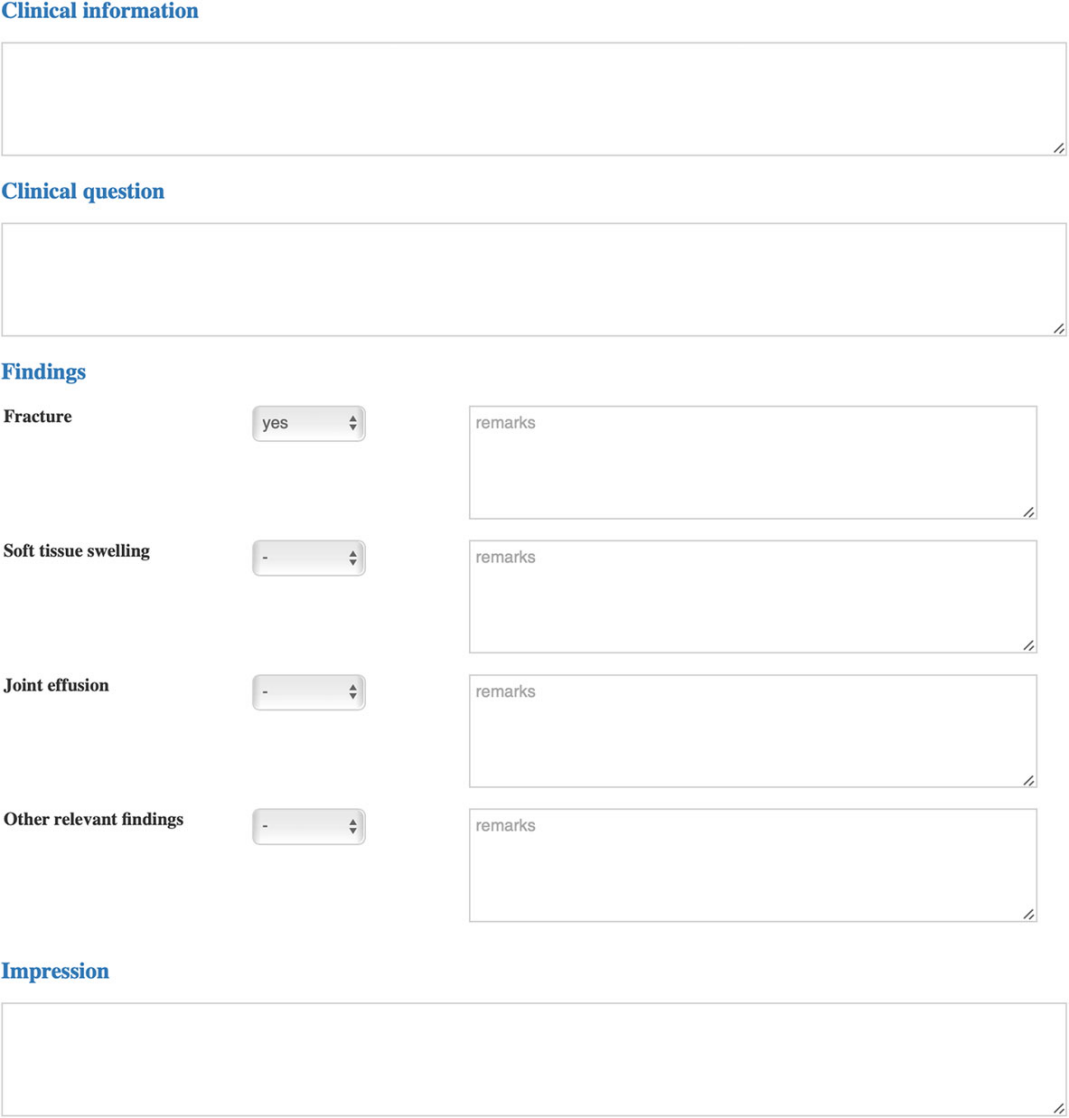
Screenshot of the template used for structured reporting of ankle radiographs

The “cx.ankle.trauma” template contained four drop-down menus where the reporting radiologist could select whether or not fracture, joint effusion, soft tissue swelling, or other relevant findings were either present or absent (Fig. 2). Apart from that, the template allowed for free-text entry of the corresponding finding. The source-code of the template can be found in Additional file 1.

**Fig. 3:**
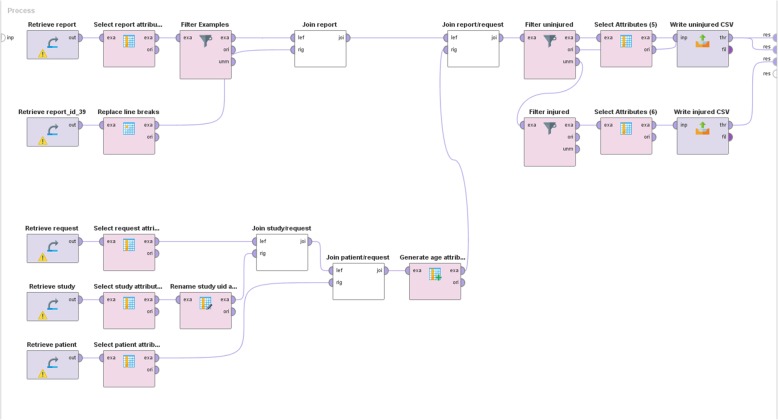
Graphical representation of the access to the report database. Various tables need to be retrieved and combined. Finally, two lists of cases with and without fractures are written and saved as CSV files

Upon completion of a report, the corresponding report content was stored in the reporting platform’s dedicated database where each report field corresponds to a specific column in the pertinent table. Consequently accessing the column “select_fracture” of the “cx.ankle.trauma” table returned either “yes” if a fracture was present or “no” if absent. Thus, we created a combination of MySQL queries that would retrieve the relevant information from the corresponding database tables. To facilitate manipulation of these data, we designed a workflow in Rapidminer 9.0 (RapidMiner, Cambridge, MA, USA) that allowed for more intuitive visualization of the data manipulation (Fig. 3). In the first step, all relevant patient and study data was queried, while also the reports created with the “cx.ankle.trauma” template were retrieved. Through joining and filtering operations, it was possible to first build a complete table where all reports were associated with the relevant patient and study information (local patient ID and DICOM Study Instance UID). Subsequently, this table was split into separate lists for reports with and without reported fractures. These lists were then exported as comma separated value (CSV) files so that in a second step a small Python (Python Software Foundation. Available at http://www.python.org) script could be used to query and retrieve the corresponding images from the institution’s PACS and export them as JPEG files into two separate folders (one folder for images with fractures and one for images without fractures).

### Convolutional neural network retraining workflow

The main focus of this study was not on the training of a convolutional neural network (CNN) but rather on the workflow of using label data from IHE-MRRT compliant report templates. We therefore chose to limit this part of the study to a simple retraining of a preexisting CNN on a binary classification task.

A TensorFlow model of the Inception V3 architecture [19], pretrained on ImageNet, was used to retrain the last fully connected layer. For the purpose of this study, we used the following standard hyperparameters: cross-entropy loss function, learning rate 0.01, batch size 32, and 2000 training steps. As the deep learning part was not the main focus, we did not attempt to optimize those settings but chose reasonable hyperparameters known to result in adequate learning performance, while also allowing for training on standard a graphics processing unit (GPU). Nevertheless, various random data augmentation techniques, such as scaling (+ 10%), cropping (− 10%), brightness (+ 10%), and horizontal flip were used to improve generalizability as the dataset was rather limited. Before retraining the CNN, 8% of all images were selected randomly and set aside from the training set to be used for validation of the final model. To compensate for unbalanced group sizes in the training dataset, the images from the smaller group were upsampled to the number of the larger group.

The computation was performed on a single server (Intel Core i7-8700K CPU, 64 GB DDR RAM, NVIDIA GeForce GTX 1080 Ti GPU). The model’s predictions and corresponding probabilities on the final validation set were recorded in a CSV file and used for calculating the diagnostic performance of the model.

### Statistical analysis

All statistical analysis was done using R 3.4.0 with RStudio 1.1.463 [20]. Receiver operating curve (ROC) analysis was performed using the pROC package [21]. To calculate sensitivity, specificity, as well as positive and negative predictive value, the operating point that yielded the highest Youden’s index was selected from the ROC analysis.

## Results

As usage of structured reporting for plain radiographs remained limited during the period included in this study (August 2017 and September 2018), only 157 out of 1186 ankle radiographs (equals to 13.2%) had been reported on by 16 different radiologists (mean reports per radiologist 10 ± 4) using the structured reporting platform.

For all of these 157 patients, anteroposterior ankle radiographs were available in the PACS and could be retrieved successfully. Mean patient age was 43.0 years (SD = 21.0 years; 76 female and 81 male). For final training and analysis, 144 images were included (129 with fractures, 28 without apparent fractures). The remaining 13 patients (eight with fractures, five without apparent fractures) were set apart as final validation set.

In order to compensate for unbalanced group sizes in the training group, the 28 images showing no fracture were upsampled (i.e., copied repeatedly) during retraining of the network to balance out the 129 images showing fractures.

**Fig. 4:**
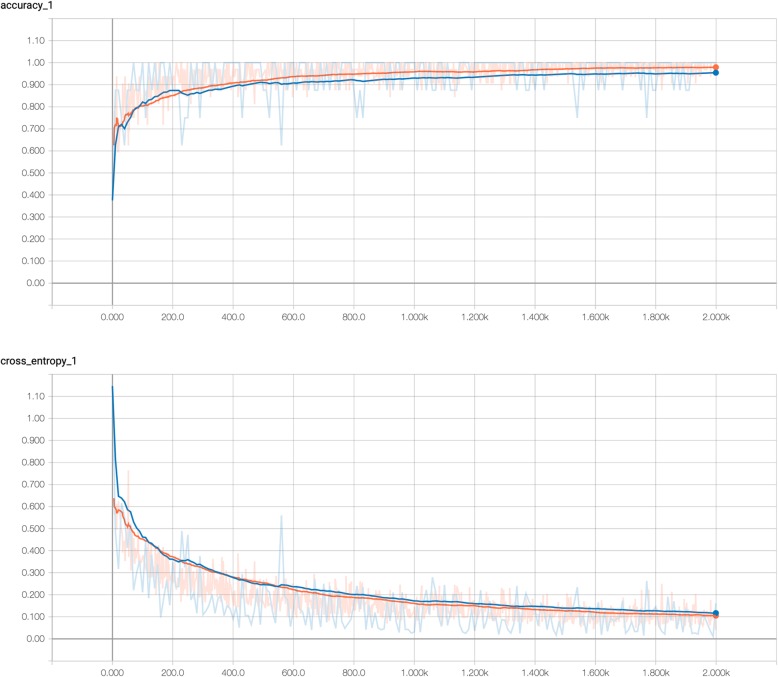
Visualization of the training process (above: accuracy, below: cross entropy, orange: training set, blue: testing set). After 2000 training steps, a final accuracy of 0.969 was achieved

Once implemented and configured, completion of the whole workflow (from database query to final evaluation of model performance) took under 1 h (retraining of the CNN accounted for around 35 min). The learning curve of the training process is shown in (Fig. 4).

**Fig. 5:**
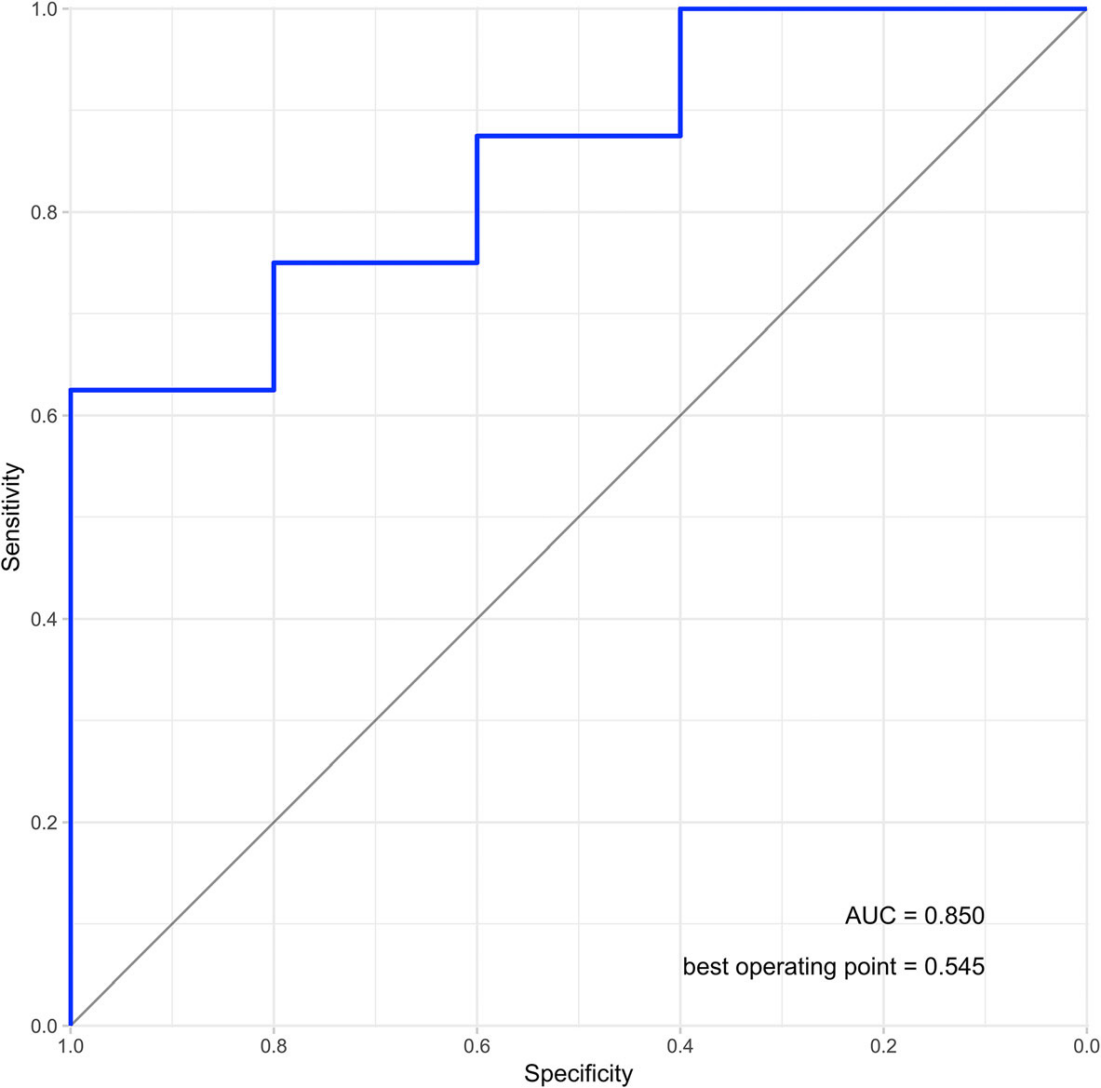
ROC-analysis for the final validation set of previously unseen images

After training, the model yielded a final accuracy (overall fraction of correct classification) of 0.769 (95% CI 0.742–0.796) on the unseen validation set (Table 1). Sensitivity was 0.625 (95% CI 0.290–1.0) and specificity 1.0 (95% CI 1.0–1.0) with a positive predictive value of 1.0 (95% CI 1.0–1.0) and a negative predictive value of 0.625 (95% CI 0.290–0.960) for presence of fracture. ROC analysis revealed an area under the curve (AUC) of 0.850 (95% CI 0.634–1.000) with an optimal operating point of 0.545 (Fig. 5).

**Table 1:** Confusion matrix showing the results on the final validation set

	Fracture (CNN)	No fracture (CNN)	Total
Fracture (true)	5	3	8
No fracture (true)	0	5	5
Total	5	8	13

## Discussion

Structured reporting has been described as the fusion reactor for radiology [22]. Various previous studies have shown that structured reports provide numerous advantages in clinical routine [23–30]. In this paper, we provide further evidence that structured reporting could play a crucial role in advancing developments in the field of radiology. Especially with the recent advent of deep learning techniques, there is a strong need for machine-readable accurate labels to images [2, 31, 32]. While many challenges of the past regarding computational power and technological issues for deep learning have been solved over the past few years, the main hurdle preventing radiology from leveraging the potential of these technologies has been a lack of large data sets with high-quality labels. This is mostly due to the fact that radiological reports are still in most cases written as unstructured narrative text. Extraction of information from such free-text reports is time-consuming and depends on the completeness and the quality of the reports. Individual variations in language and style can lead to inconsistencies and uncertainties that could potentially impair the quality of the dataset. Therefore, researchers need to rely on manually reviewing and labeling data, which can be time-consuming and is therefore difficult to implement on a large scale. Theoretically, these challenges could be overcome by using natural language processing (NLP) to extract the relevant information from the radiological reports. However, this can potentially introduce a relevant number of incorrect labels to the dataset since generally sensitivity and specificity of such systems are only around 90% [33].

Our proposed workflow addresses these challenges since it utilizes data from structured reports generated during routine clinical practice. Thus, no additional workup of the dataset is needed to provide reliable and standardized labels for the training of deep learning algorithms. Considering that only a rather small fraction (13.2%) of all reports was created using the structured reporting templates during the period included in this study, it can be assumed that the pe-rformance of the trained model could substantially be improved if more radiographs would have had corresponding structured reports. Certainly, the most important challenge radiologists face when using structured reporting is the notable change in workflow. In our case, the structured reporting platform required the user to use the mouse and the keyboard to input the report, thus preventing him from work with the PACS viewer while composing the report. Better integration of structured reporting tools (e.g., with speech recognition and tighter PACS integration) could help to improve the adoption of structured reporting in clinical routine.

The present study has some limitations: first, we did not re-evaluate the reports for diagnostic accuracy. Secondly, and certainly more importantly, the dataset used for the purpose of this study was rather small and unbalanced. There are several options to address such imbalances. In our case, we opted to apply oversampling of the underrepresented class (no fracture) as we did not want to discard any useful data. However, this approach has a certain tendency to overfit, since some examples are used multiple times. To alleviate this effect, we applied data augmentation techniques to the training dataset (scaling, flipping, cropping, etc.). Nevertheless, for a clinically applicable algorithm, other solutions to the class imbalance problem should be considered, such as undersampling, cost-sensitive learning, or other more advances techniques [34–36].

Performance of the algorithm therefore needs to be viewed as only preliminary and not clinically useful, especially since a selection bias toward simple cases in which the radiologists were more comfortable using the structured reporting platform cannot be ruled out. However, this was beyond the intended scope of this study. The proposed workflow nevertheless clearly demonstrates and underlines the value of structured reporting in the context of machine learning and artificial intelligence and is in line with the key research priorities as defined by in an intersociety roadmap for foundational research on artificial intelligence in medical imaging [37, 38]. Especially with the possibilities to link specific parts of the report content to ontologies such as RadLex, the IHE MRRT profile provides an interoperable way to allow for easier pooling of datasets across various institutions while maintaining reliable label data [18, 39].

## Conclusion

Of course, a widespread implementation of structured reporting will have a significant impact on the radiologist’s daily work and may not be applicable to all cases and all clinical scenarios. Nevertheless, our study further highlights the need for to push toward more structured reporting in clinical routine, as it seems the most practical approach to obtain high-quality report data for various future developments. Users should therefore urge vendors to provide practical solutions that allow for easy access to and usage of report information for further analysis and usage in deep learning projects.

## Additional file

Additional file 1: cx.ankle.trauma template. (HTML 4 kb)

## Data Availability

The datasets used and/or analyzed during the current study are available from the corresponding author on reasonable request.

## Abbreviations

AUC: Area under the curve
CNN: Convolutional neural network
CPU: Central processing unit
CSV: Comma separated values (a file format)
FDA: Food and drug administration
IHE MRRT: Integrating the healthcare enterprise management of radiological report templates
GPU: Graphics processing unit
JPEG: Joint photographic experts group (a file format)
MySQL: My structured query language (a database management system)
NLP: Natural language processing
PACS: Picture archiving and communication system
RIS: Radiology information system
ROC: Receiver operating curve
SD: Standard deviation
