# Supplementary material for: Structured report data can be used to develop deep learning algorithms: a proof of concept in ankle radiographs
Source: Insights Imaging. 2019 Sep 23;10:93. doi: 10.1186/s13244-019-0777-8 (PMC6777645; doi:10.1186/s13244-019-0777-8)
Supplement: Supplementary file 1 — cx.ankle.trauma template. (HTML 4 kb) [file 13244_2019_777_MOESM1_ESM.html]

ankle radiograph (trauma)


Clinical information

Clinical question

Findings

|  |  |  |
| --- | --- | --- |
| Fracture | - yes no |  |

|  |  |  |
| --- | --- | --- |
| Soft tissue swelling | - yes no |  |

|  |  |  |
| --- | --- | --- |
| Joint effusion | - yes no |  |

|  |  |  |
| --- | --- | --- |
| Other relevant findings | - yes no |  |

Impression
